# Neuroinflammation mediates the progression of neonate hypoxia-ischemia brain damage to Alzheimer’s disease: a bioinformatics and experimental study

**DOI:** 10.3389/fnagi.2024.1511668

**Published:** 2025-01-13

**Authors:** Shengjie Zhang, Ruqiu Zhang, Zhaoqin Chen, Zihan Shao, An Li, Fan Li, Fang Huang

**Affiliations:** ^1^School of Medicine, Yunnan University, Kunming, China; ^2^State Key Laboratory for Conservation and Utilization of Bio-resources in Yunnan, Yunnan University, Kunming, China; ^3^Changxin School, Yunnan University, Kunming, China; ^4^Medical College, Shantou University, Shantou, China

**Keywords:** Alzheimer’s disease, neonate hypoxia-ischemia brain damage, bioinformatics analysis, neuroinflammation, pathophysiological mechanisms

## Abstract

**Background:**

Traumatic brain injury (TBI) can generally be divided into focal damage and diffuse damage, and neonate Hypoxia-Ischemia Brain Damage (nHIBD) is one of the causes of diffuse damage. Patients with nHIBD are at an increased risk of developing Alzheimer’s disease (AD). However, the shared pathogenesis of patients affected with both neurological disorders has not been fully elucidated.

**Purpose:**

We here aim to identify the shared molecular signatures between nHIBD and AD. We used an integrated analysis of the cortex gene expression data, targeting differential expression of genes related to the mechanisms of neurodegeneration and cognitive impairment following traumatic brain injury.

**Methods:**

The gene expression profiles of Alzheimer’s disease (GSE203206) and that of Neonate Hypoxia-Ischemia Brain Damage (GSE23317) were obtained from the Gene Expression Omnibus (GEO) database. After identifying the common differentially expressed genes (DEGs) of Alzheimer’s disease and neonate Hypoxia-Ischemia Brain Damage by limma package analysis, five kinds of analyses were performed on them, namely Gene Ontology (GO) and pathway enrichment analysis, protein–protein interaction network, DEG-transcription factor interactions and DEG-microRNA interactions, protein-drug interactions and protein-disease association analysis, and gene-inflammation association analysis and protein-inflammation association analysis.

**Results:**

In total, 12 common DEGs were identified including HSPB1, VIM, MVD, TUBB4A, AACS, ANXA6, DIRAS2, RPH3A, CEND1, KALM, THOP1, AREL1. We also identified 11 hub proteins, three central regulatory transcription factors, and three microRNAs encoded by the DEGs. Protein-drug interaction analysis showed that CYC1 and UQCRFS1 are associated with different drugs. Gene-disease association analysis shows Mammary Neoplasms, Neoplasm Metastasis, Schizophrenia, and Brain Ischemia diseases are the most relevant to the hub proteins we identified. Gene-inflammation association analysis shows that the hub gene AREL1 is related to inflammatory response, while the protein-inflammation association analysis shows that the hub proteins AKT1 and MAPK14 are related to inflammatory response.

**Conclusion:**

This study provides new insights into the shared molecular mechanisms between AD and nHIBD. These common pathways and hub genes could potentially be used to design therapeutic interventions, reducing the likelihood of Alzheimer’s disease development in survivors of neonatal Hypoxic-Ischemia brain injury.

## 1 Introduction

Traumatic brain injury (TBI) is one of the major causes of disability and death worldwide. The damages from TBI can generally be divided into focal damage and diffuse damage. Diffuse injuries include hypoxia-ischemic injury, meningitis, and vascular injury. Neonate Hypoxia-Ischemia Brain Damage (nHIBD) is one of the causes of TBI and occurs at the beginning of human life. It is a neurological condition because neonatal hypoxia and ischemia lead to the dysfunction and loss of neuronal, glial, and endothelial cells, which are vital in the human brain. In recent years, the advancement of nHIBD treatment methods has included hypothermia, stem cell therapy, and neuroprotective drugs (Wu et al., 2015; Yang et al., 2020). While hypothermia is a widely acceptable treatment, there are still some neonatal patients suffering from long-term neurological dysfunction after being treated. About 0.7% of premature infants and 0.3% of full-term infants suffer from neonatal hypoxia ischemic encephalopathy, among whom only 60% survive, and 30% of survivors will suffer from neurological dysfunction, including Alzheimer’s disease (AD), in the long term (Douglas-Escobar and Weiss, 2015; Kurinczuk et al., 2010).

AD is the leading form of neurodegenerative disease, mostly affecting the elderly and characterized by a gradual deprivation of cognitive function, ultimately leading to death (Alzheimer’s Association et al., 2017). The Symptoms of AD include depression, repetitive questioning (Huang et al., 2020), aggression, sleep problems, wandering, and a variety of inappropriate behaviors, and 95% of AD occurs sporadically. Postmortem tissue analysis of AD brains revealed the existence of Aβ amyloid plaques and neurofibrillary tangles of hyperphosphorylated Tau (Huang et al., 2019). These constitute the two hallmarks of AD and have distinct features.

It has been reported that patients who experienced neonatal hypoxia-ischemia encephalopathy are more prone to AD than their peers (Tarkowska, 2021), but the exact mechanism by which these two diseases coexist remains unclear. Interestingly, both nHIBD and AD have been demonstrated to be associated with TLR4 and Ferroptosis, and AQP4 level was reported to be decreased in both nHIBD and AD (Chandra et al., 2021; Ruganzu et al., 2022; Wang et al., 2023; Yu et al., 2012; Zhu et al., 2021). However, there are still insufficient bioinformatics studies investigating the relationship between nHIBD and AD. The study of common transcriptional features shows promise as a novel and feasible scheme for investigating the common pathogenic mechanisms of nHIBD and AD at the genetic level. Therefore, the primary aim of this study is to explore the common pathophysiological mechanisms of AD and nHIBD. To achieve our objectives, we analyzed two gene expression datasets (GSE23317 and GSE203206) downloaded from the Gene Expression Ontology (GEO) database to identify the differentially expressed genes (DEGs) for the two datasets, and then the common DEGs genes for the two diseases. The identified common DEGs are the focus of this study and thus are used for further experiments and analyses including:

Gene Ontology (GO) and pathway enrichment analysis to find shared GEGs and pathways;
Construction of protein–protein interaction networks (PPIs) with the STRING database and their analyses with the Cytoscape software to find hub protein;
Analyses of the DEG-transcription factor interactions and DEG-microRNA interactions;
Analyses of the protein-drug and protein-disease interaction network to find potential drugs and potential treatment methods by identifying the transcriptional components of universal DEGs;
Analyses of gene-inflammation and protein-inflammation association to find the relationship between DEGs and inflammation responses.

**Figure 1:**
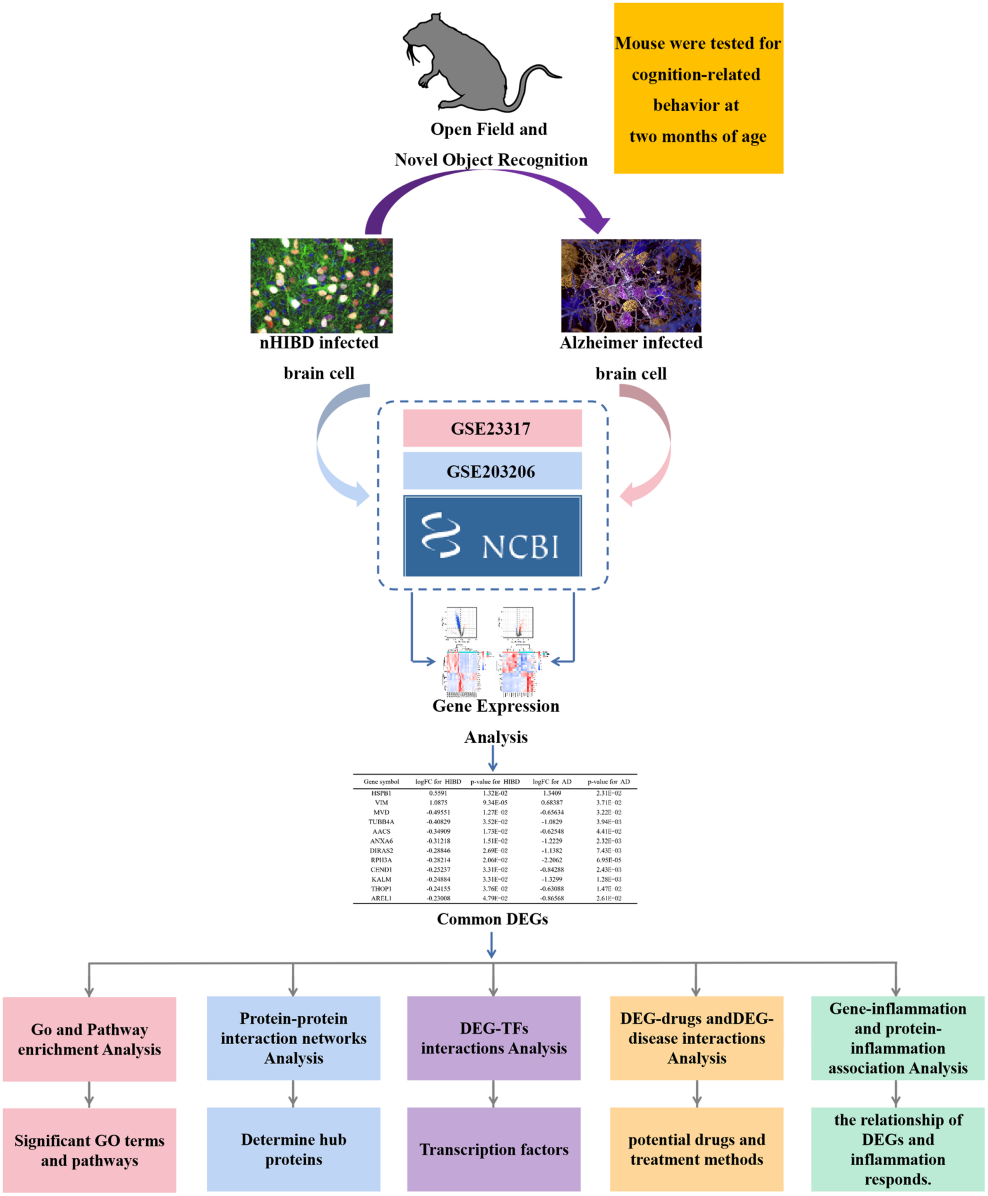
Flowchart of the analytical steps of this study. DEGs, differentially expressed genes; GO, Gene Ontology; TF, Transcription factor; miRNA, microRibonucleic acid.

Therefore, the main objective of this study was to construct a workflow based on bioinformatics approaches to detect potential associations between nHIBD and AD. Determining the nature of these associations may not only provide clues to the molecular processes underlying these diseases but also ultimately help to discover possible treatments that could lead to the generation of disease-modifying drugs. Figure 1 illustrates the sequential workflow of our study.

## 2 Materials and methods

### 2.1 Data source

GEO^1^ is a public database that includes a large number of high-throughput sequencing and microarray gene sets (Edgar et al., 2002). Using Alzheimer’s disease (AD) and neonate hypoxia-ischemia brain damage (nHIBD) as keywords, the related gene expression datasets were exhibited and two microarray datasets (GSE23317 and GSE203206) from the cortex genome from GEO (Illumina MouseRef-8 v2.0 GPL6885 platform, Illumina HiSeq 4,000 GPL20301 platform) were downloaded. The GSE23317 dataset contains the genome of neonatal mice aged 8 days with nHIBD treated by Rice-Vannucci (*n* = 7) and sham control (*n* = 8) from the cortex. The dataset was processed using high-throughput functions analyzed by Illumina Miseq (*Mus musculus*) and contributed by Cheung et al. (GEO: GSE23317). GSE203206 consists of the genome from the occipital cortex of patients with early AD (*n* = 19) and healthy controls (*n* = 8). The dataset is processed using high-throughput functions of the analysis using Illumina HiSeq (*Homo sapiens*). This is from a database contributed by Caldwell et al. (2022) and Scheckel et al. (2016).

### 2.2 Identification of DEGs

Differentially expressed genes (DEGs) were identified using the “Limma” package of R software (Ritchie et al., 2015). Meanwhile, the DEGs of the GSE203206 dataset had a threshold | logFC | > 0.5 and *p*- value <0.05, and the DEGs of the GSE23317 dataset had a threshold | logFC | > 0.2 and *p*- value <0.05. The Funrich analysis tool was used to obtain the common DEGs between AD and nHIBD.

### 2.3 Gene ontology and pathway enrichment analysis

To help understand the function of differentially expressed genes, GO function, KEGG (Kyoto Encyclopedia of Genes and Genomes) analysis, and pathway enrichment analysis were carried out using DAVID software (version 2023q2). GO is a multifaceted annotation of the genome for biological processes, cellular components, and molecular functions. At the same time, the KEGG annotates the genetic pathways of different species in many ways, providing information about biological functions (Masseroli, 2019). Both the GO and KEGG pathways were enriched by the omicshare database. The data we processed used Fisher’s exact test as a statistical method and was corrected by the Benjamini algorithm, with *p* < 0.05 after correction being considered as a significant difference.

### 2.4 Protein–protein interaction network analysis

In all living organisms, PPIs are critical for both cellular functions and biological processes. Exploration of protein interactions will facilitate a deeper understanding of pathophysiological pathways of disease conditions as well as the identification of multiple drugs and therapeutically optimized methods (Rain et al., 2001). Using the protein interaction gene retrieval tool^2^ (version 11.5), the minimum required interaction score was set to 0.4 to mine the interactions between protein-coding genes, relationships between proteins of interest, such as direct binding or coexisting upstream and downstream regulatory pathways could be found, and can help to build a PPI network with complex regulatory relationships to reflect our specified DEGs and how proteins can physically and functionally communicate with each other (Szklarczyk et al., 2019). Due to the relatively small number of common DEGs, the lowest score confidence criterion was set to generate the PPI network for the analysis using network analysis.

Three densely connected modules from the PPI network were identified with the criteria of K-core = 2, degree cutoff = 2, max depth = 100, and node score cutoff = −0.2 by the MCODE plugin of Cytoscape (versions 3.8.0).

### 2.5 Identification of transcription factors and miRNA with common genetic interactions

NetworkAnalyst tool^3^ was used to construct the DEG-transcription factor interactions and DEG-microRNA interactions (Xia et al., 2015). Finally, these hub genes, miRNA, and transcription factor were plotted using the NetworkAnalyst tool.

### 2.6 Assessment of the protein-drug interactions

DrugBank (v5.0) is a unique bioinformatics and cheminformatics resource, which also provides information about the effects of drugs on expressed proteins. Network analysis was utilized for protein-drug interactions to find interactions between the identified DEGs and drugs in the drug library dataset.

### 2.7 Gene-disease association assessment

DisGeNET is a standardized database of gene-disease associations, which integrates relationships from multiple sources involving different biomedical aspects of the disease and has demonstrated a growing understanding of the human genetic disease. Taking advantage of DisGeNET, network analysis was used to examine gene-disease interactions to identify disease and chronic complications associated with DEGs (Piñero et al., 2017).

### 2.8 Gene-inflammation and protein-inflammation association analysis

UniProt Knowledgebase^4^ is a database that provides users with a comprehensive, high-quality, and freely accessible set of protein sequences annotated with functional information (UniProt Consortium et al., 2021). This database was used to analyze each DEG and hub protein we have identified to find the DEG and hub proteins related to the inflammatory response.

### 2.9 Establishment of a neonatal mouse model of HIBD

Given that most studies have adopted the Rice-Vannucci method which mimics neonatal encephalopathy in term infants (Min et al., 2020; Rice et al., 1981), this method was used to establish the nHIBD mouse model in this study. Pregnant C57BL/6 J mice (aged >15 days of gestation) were procured from the Animal Center of Yunnan University (Kunming, China), All the animals were acclimated under standard laboratory conditions (ventilated room, 25 ± 1°C, 60 ± 5% humidity, 12 h light/dark cycle) and had free access to standard water and food (SCXKK2021-0001). All procedures were conducted in accordance with the “Guiding Principles in the Care and Use of Animals” (China) and were approved by the Laboratory Animal Welfare Ethics Committee Yunnan University (YNU20230470). The obtained C57BL/6 J mice were fed under standardized environmental conditions. After delivery, the pups were anesthetized with isoflurane on postnatal days 9–11 (P9-P11) (4% for induction and 2% for Supplementary material, anesthesia). The artery was cut on the middle skin of the mouse neck, the left carotid artery around the trachea was identified and the left common carotid artery was isolated. Subsequently, double ligation was performed with suture (6.0) and the neck skin was immediately closed. The whole surgical procedure was controlled within 5 min. The pups were then returned to their mother for 1 h. Next, the pups were placed in a hypoxic chamber (8% O_2_ + 92% N_2_ humidified) for 40 min and placed in a hypoxic environment to induce hypoxic damage. After completion of the hypoxic insult, the mice were placed around their mother. Mice receiving anesthesia and carotid artery exposure but without ligation were used as controls (Sham).

### 2.10 Open field test

**Figure 2:**
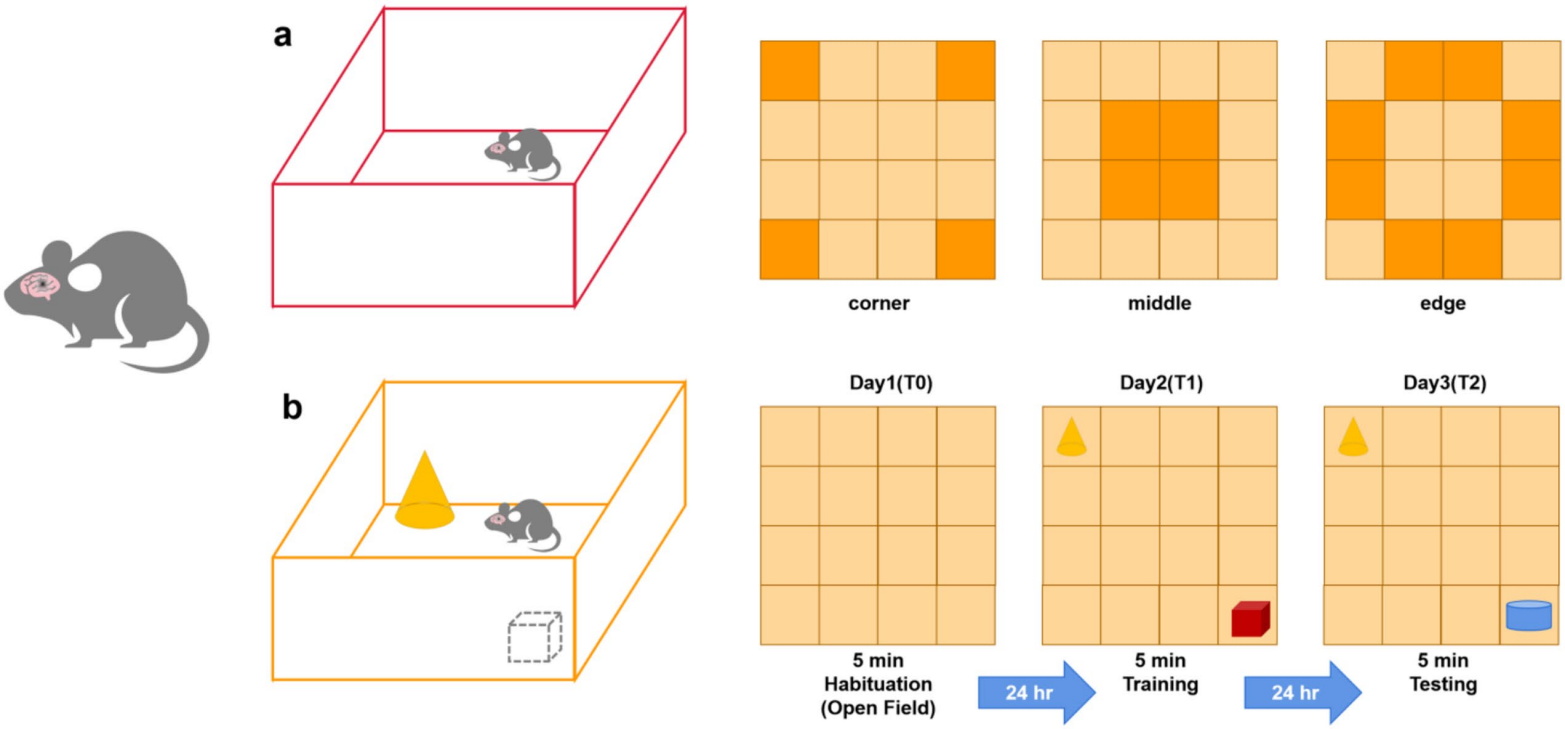
Patterns of behavioral experiments in mice. **(A)** Open Field test, divide white opaque box into 4*4, the side area including corner area and edge area, the center area including middle area; **(B)** Novel Object Recognition, divide white opaque box into 4*4, place different objects in the diagonal area (in the center of corner area).

Two months after the nHIBD model was established, these mice were individually tested in a 45*45*45 cm^3^ white opaque box with the room temperature kept at 25°C. After each trial, the box was cleaned with 75% alcohol. Each test lasted 5 min, and the mice were allowed to move freely inside the box while the data were collected by video system, VisuTrack (China) after 5 min of adaptation (Kraeuter et al., 2019; Figure 2A). The box was equally divided into 16 grids and 3 parts including the middle, edge, and corner. Then we focused on the duration of time they spent in different areas, and we evaluated their anxiety level by their frequency of standing, urinating and defecating, face scratching, and wall climbing. When the data were normally distributed, we performed a difference analysis using an independent-samples t-test. When the data do not conform to the normal distribution, we perform a difference test using a nonlinear analysis.

### 2.11 Novel object recognition test

The Novel Object Recognition test was performed 1 day after the Open Field test in the same place. It consisted of the following phases: an adaptation phase, training phase (a familiar object A and a familiar object B), and testing Phase (a familiar object A and an unfamiliar object C) (Faraco et al., 2019). Episodic memory was tested at 5 min (training phase) after the adaptation phase and another 5 min (testing Phase) after 24 h. Then the number of times the animal touched the two objects and the time spent on the two objects were recorded. The index of novel object recognition was calculated by the following formula: The time of contact with the new object on the next day was divided by the sum of the time of contact with the two objects, or the number of contacts with the new object was divided by the sum of the number of contacts with the two objects (Figure 2B). Then the results were statistically analyzed to judge the short-term learning and memory ability of the mice. When the data were normally distributed, we performed a difference analysis using an independent-samples t-test. When the data do not conform to the normal distribution, we perform a difference test using a nonlinear analysis.

### 2.12 RT-qPCR

Total RNA content was extracted from the mouse cortex using the TRIzol reagent (15596018CN, ThermoFisher Scientific, Rockford, United States). The RNA concentration was measured by a microplate reader (DG-3022 type A). The cDNA was obtained from RNA by reverse transcription with a reverse transcription kit (AQ601, TransGen Biotech, Beijing, China). The resulting amount of cDNA was 2 μL and was mixed with SYBR Premix Ex Taq (AQ601, TransGen Biotech, Beijing, China) to obtain a standard 20 μL PCR reaction mixture. In the Applied Biosystems real-time PCR system, the annealing temperature was set to perform a real-time quantitative PCR reaction, which was later analyzed according to the final data. The sequences of the quantitative PCR primers used in this study are shown in Table 1. When the data were normally distributed, we performed a difference analysis using an independent-samples t-test. When the data do not conform to the normal distribution, we perform a difference test using a nonlinear analysis.

**Table 1:** Primers employed in this study.

Gene	Forward sequence	Reverse sequence
VIM	CGGCTGCGAGAGAAATTGC	CCACTTTCCGTTCAAGGTCAAG
DIRAS2	CTCTCCAGCAACATGCCTGA	CACTTGCCGGTAGGTGTCTT
RPH3A	ACTGTGGTGAACCGATGGATG	CCTCGTCTGTCAGTTCTTCCTG
AREL1	TCTGTCATCTGCAAGCCCTG	GGACCAACGAAGTGACAGCA

## 3 Results

### 3.1 Identification of DEGs

**Figure 3:**
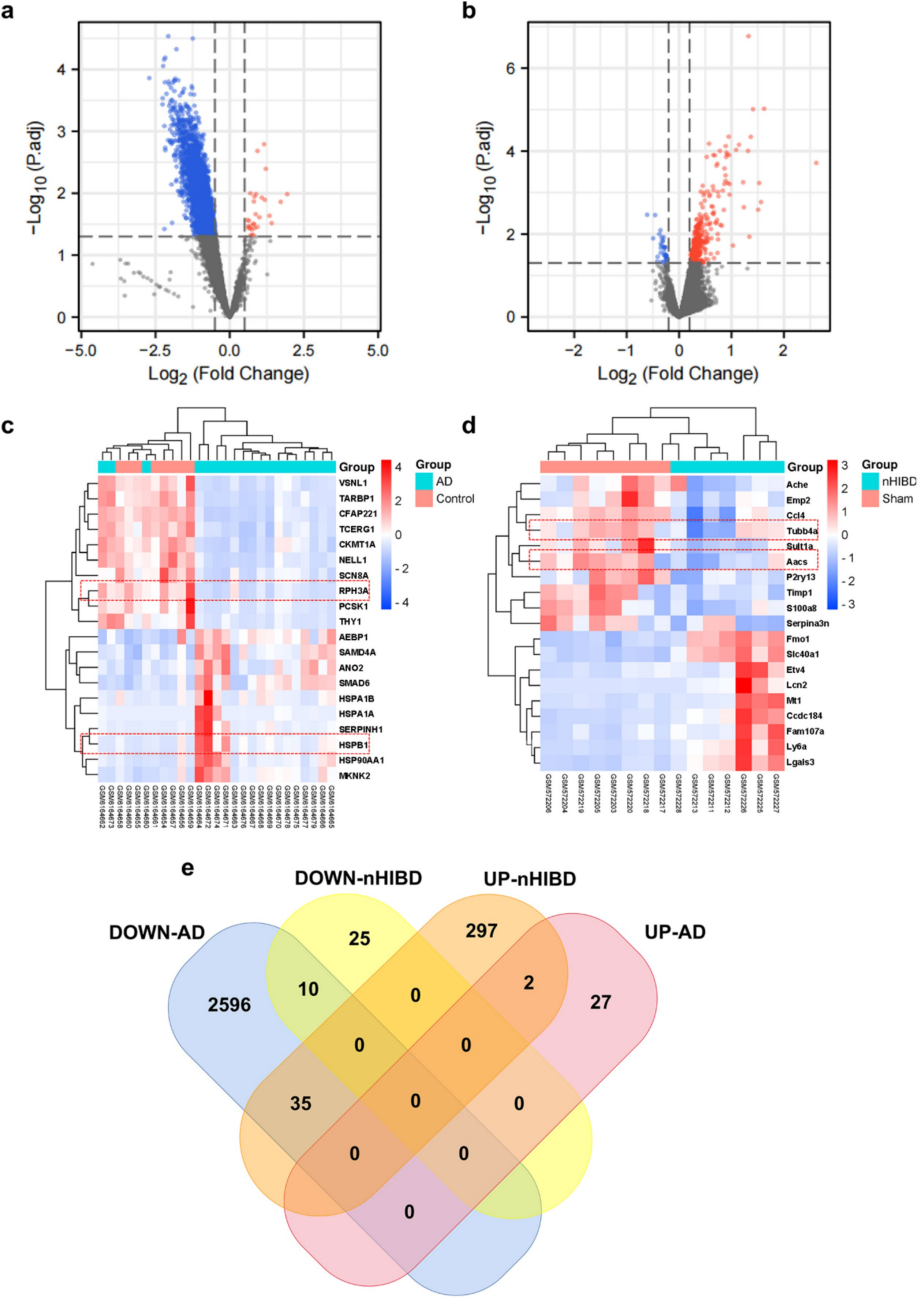
Microarray normalization and differential gene analysis in the AD and nHIBD group. **(A)** The volcano map of AD, blue represents downregulation, and red represents upregulation, | logFC | > 0.5 and *p*-value <0.05. **(B)** The volcano map of nHIBD, blue represents downregulation, and red represents upregulation, | logFC | > 0.2 and *p*-value <0.05. **(C)** The heatmap of AD. **(D)** The heatmap of nHIBD. **(E)** The two datasets showed an overlap of 12 DEGs.

In the nHIBD dataset, we analyzed 369 differentially expressed genes at 8 h and 24 h after nHIBD, out of which 334 were up-regulated genes and 35 were down-regulated. In the AD dataset, we identified 2,670 significant DEGs, with 29 DEGs substantially up-regulated and 2,641 DEGs substantially down-regulated. In total, 12 common DEGs (10 down-regulated, 2 up-regulated) were obtained after excluding genes with opposite expression trends between GSE23317 and GSE203206. Although the differential genes of neonatal hypoxia-ischemia are mainly upregulated, and those of AD are mainly downregulated, they share very few common differentially expressed genes with consistent trends. However, this small number of shared differentially expressed genes plays an important role in both conditions (Figure 3), including *HSPB1, VIM, MVD, TUBB4A, AACS, ANXA6, DIRAS2, RPH3A, CEND1, KALM, THOP1, AREL1, and HSPB1, VIM* are upregulated while others are downregulated.

### 3.2 Analysis of the functional features of common DEGs

**Figure 4:**
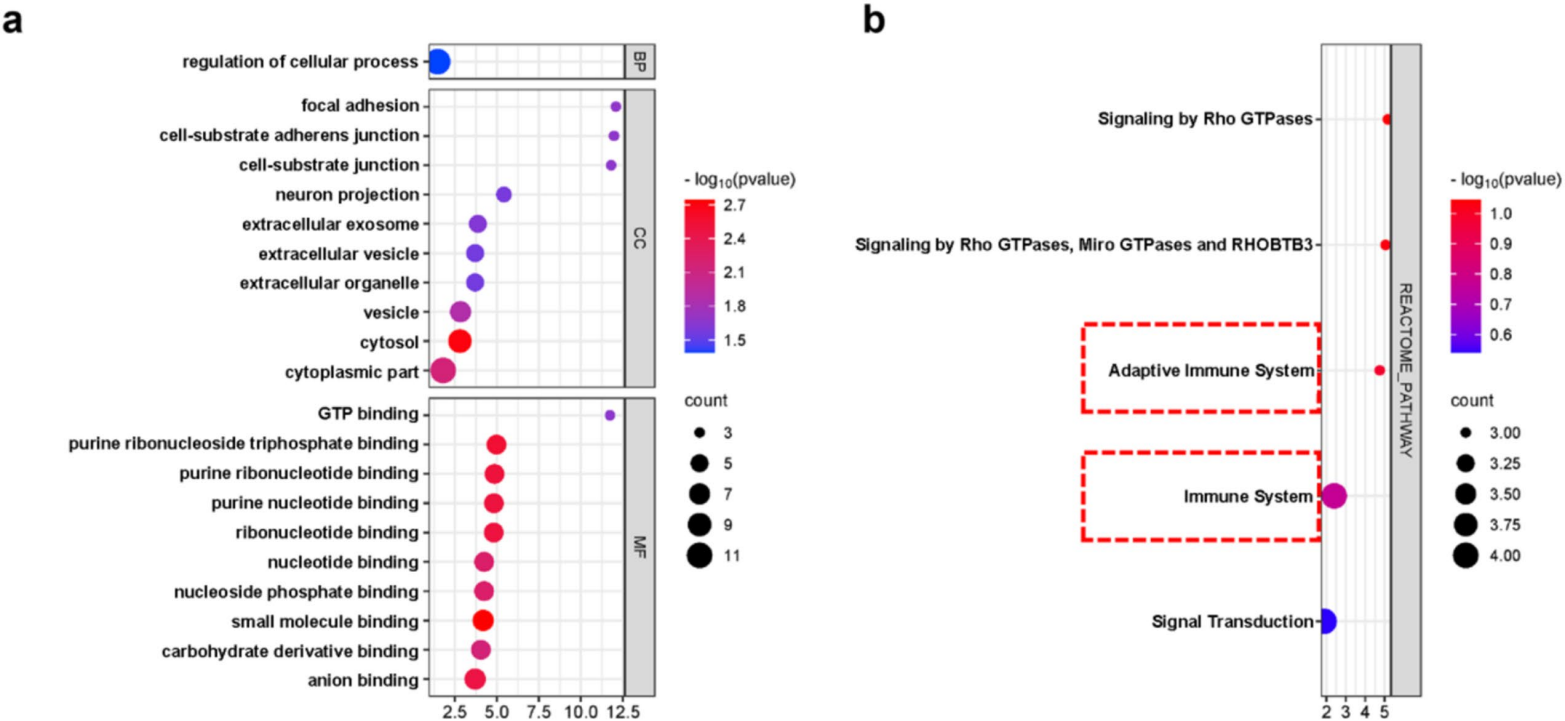
DEGs enrichment analysis results. **(A)** The enrichment analysis results of GO, adjusted *p*-value <0.05 was considered significant. **(B)** The enrichment analysis results of the KEGG Pathway. X-axis is fold enrichment.

In order to clarify the biological functions and pathways involved in the progression of nHIBD to AD, GO functions and KEGG Pathway enrichment analyses were performed to analyze the 12 common DEGs (Table 2). GO analysis results revealed that the common DEGs are involved in 1 biological process (BP), which is the regulation of cellular process; 24 cellular components (CC), including cell-substrate adherent junction, cell-substrate junction, focal adhesion, neuron projection, and extracellular exosome; 19 molecular functions (MF), such as GTP binding, small molecule binding, purine ribonucleoside triphosphate binding, purine ribonucleotide binding, and nucleotide binding (Figure 4A). Nucleotide-binding oligomerization domain-like receptor protein 3 (NLRP3) is a inflammasome, associated with AD and nHIBD, as well as nucleotide binding (Chen et al., 2023a; Heneka et al., 2013). The results of the pathway enrichment analysis showed that these genes are involved in signaling by Rho GTPases, Miro GTPases, and RHOBTB3, signal transduction, immune system and adaptive immune system (Figure 4B). Since B-cell is an important part of the adaptive immune system, which plays an important role in the pathogenesis of various central nervous system (CNS) diseases, and B cells mediate autoimmune inflammation in the CNS through the release of inflammatory factors (Sabatino et al., 2019). The inductive inference is that inflammatory is involved in the progression of nHIBD to AD.

**Table 2:** LogFC and *p*-values for each common DEGs.

Gene symbol	logFC for nHIBD	p-value for nHIBD	logFC for AD	*p*- value for AD
*HSPB1*	0.5591	1.32E-02	1.3409	2.31E-02
*VIM*	1.0875	9.34E-05	0.68387	3.71E-02
*MVD*	−0.49551	1.27E-02	−0.65634	3.22E-02
*TUBB4A*	−0.40829	3.52E-02	−1.0829	3.94E-03
*AACS*	−0.34909	1.73E-02	−0.62548	4.41E-02
*ANXA6*	−0.31218	1.51E-02	−1.2229	2.32E-03
*DIRAS2*	−0.28846	2.69E-02	−1.1382	7.43E-03
*RPH3A*	−0.28214	2.06E-02	−2.2062	6.95E-05
*CEND1*	−0.25237	3.31E-02	−0.84288	2.43E-03
*KALM*	−0.24884	3.31E-02	−1.3299	1.28E-03
*THOP1*	−0.24155	3.76E-02	−0.63088	1.47E-02
*AREL1*	−0.23008	4.79E-02	−0.86568	2.61E-02

### 3.3 Construction of PPI networks and identification of hub proteins

**Figure 5:**
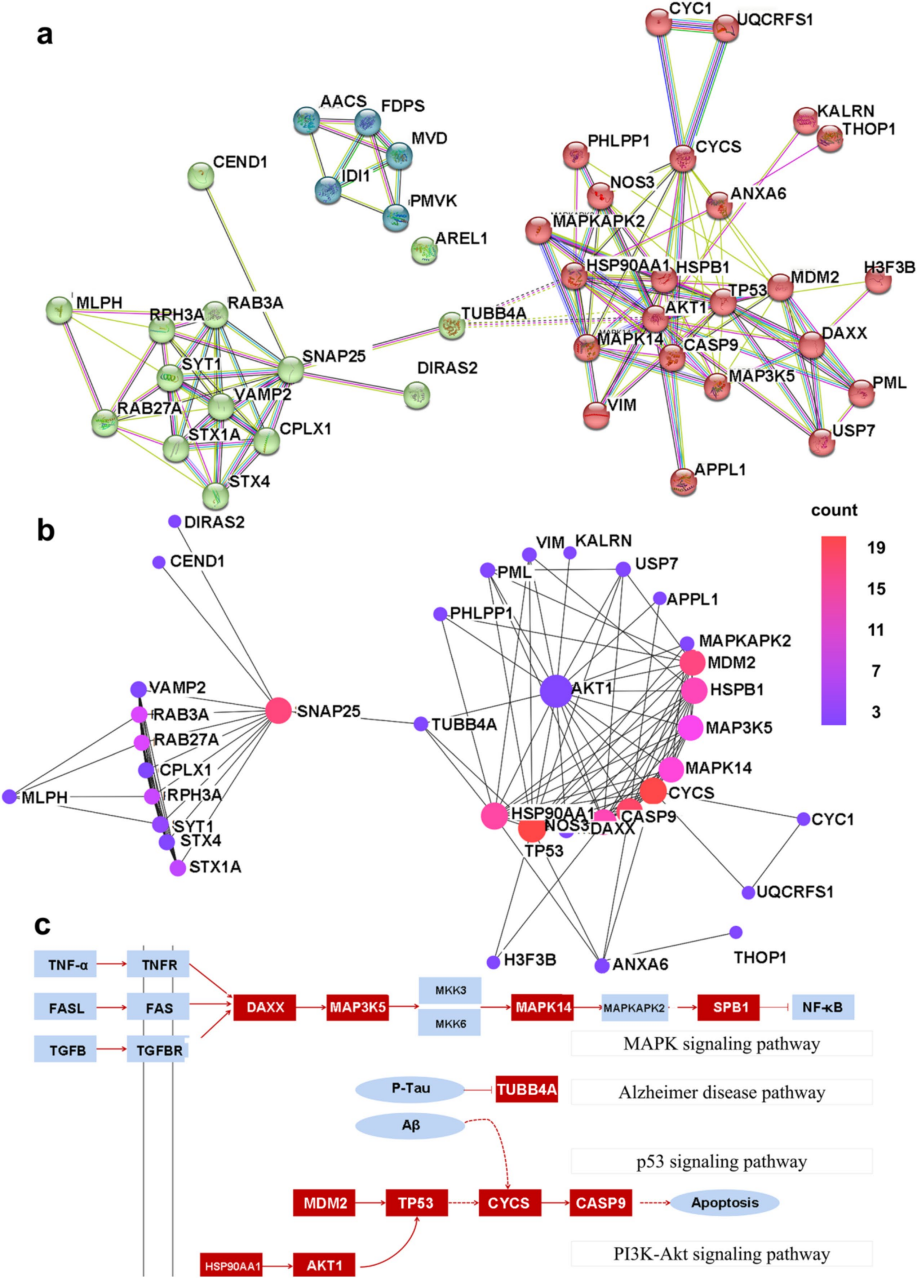
The PPI network map. **(A)** The shared PPI network map by nHIBD and AD. **(B)** The hub protein in the PPI network, and the color scale is points edge count. **(C)** The relationship between DEGs and hub protein, and the red parts are DEGs or hub protein.

PPI networks were constructed for DEGs with a combined score greater than 0.4 using Cytoscape, and this resulted in 42 nodes and 131 edges. In addition, three closely connected gene modules were obtained by the MCODE plug-in of Cytoscape (Figure 5A). Several interconnected nodes in a PPI network are considered to be hub proteins. By studying the Cytohubba plug-in results of Cytoscape identified with different algorithms, a total of 11 hub proteins were detected, including AKT1, TP53, CYCS, HSPB1, CASP9, SNAP25, MDM2, MAPK14, MAP3K5, DAXX, and HSP90AA1 (Figure 5B).

### 3.4 Identification of transcription factors and miRNA with common genetic interactions

**Figure 6:**
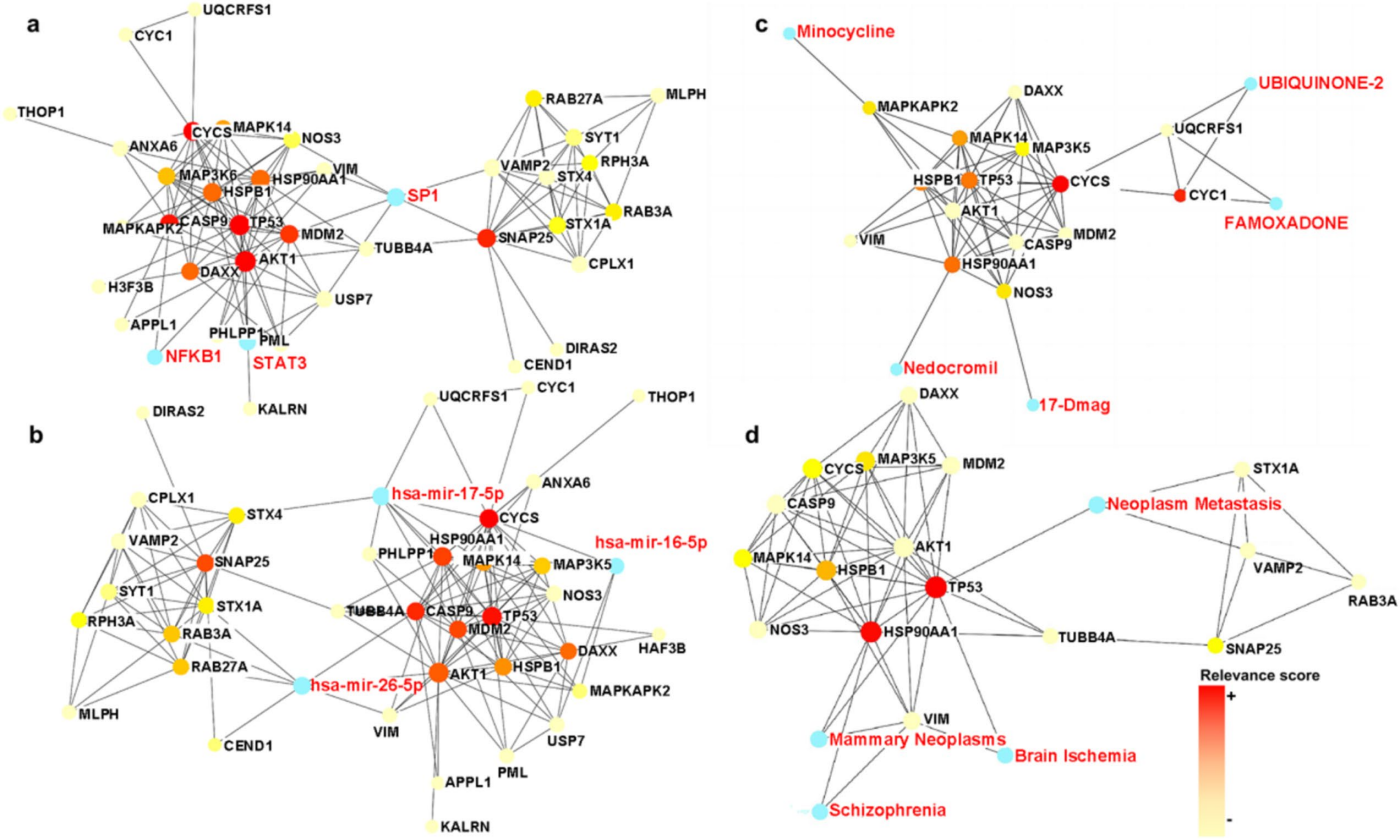
TFs and miRNA plots and interaction plot between the drugs and the hub proteins. **(A)** The Gene-TFs interactions, and the blue parts are key transcription factors. **(B)** The Gene-miRNA interactions, and the blue parts are key miRNA. **(C)** Interaction plot between the drugs and the hub proteins, and the blue parts are key brugs. **(D)** Interaction plot between the disease and the hub proteins, and the blue parts are associated diseases, the color scale is points relevance score. TF, Transcription factor; miRNA, microRibonucleic acid.

PPI-based methods were used to read the transcription factors (TF), miRNAs, DEGs-TFs, and DEGs-miRNA to identify transcriptional and post-transcriptional regulation of the common DEGs. The association involving the DEGs and the TFs is shown in Figure 6A. The correlation between DEGs and miRNAs is also shown in the figure, and this identified three transcription factors including STAT3, SP1, and NFkB. Among them, STAT3 and NFkB are involved in the classical pathway linking inflammation and cancer. S-sulfhydration of Stat3 attenuates osteopontin-mediated neuroinflammation following hypoxia-ischemia insult in neonatal mice (Li et al., 2022). STAT3 acetylation and activation plays an important part in astrocytic mitochondrial dysfunction progressively induces neuroinflammation and AD (Mi et al., 2023). miR-212-3p attenuates neuroinflammation of rats with AD via regulating the SP1/BACE1/NLRP3/Caspase-1 signaling pathway (Nong et al., 2022). nHIBD activated the NF-kB signaling and NF-κB-mediated cytokine, modulating Inflammatory Response (Sun et al., 2022). NF-kB (p50/p65)-Mediated Pro-Inflammatory microRNA (miRNA) Signaling in AD (Lukiw, 2022). The analysis also identified three miRNAs including mir-17-5p, mir-26b-5p, and mir-16-5p (Figure 6B), among which mir-17-5p and mir-16-5p are associated with tumors, indicating that there are some links between miRNA enriched by DEGs and tumors and inflammation. What’s more, miR-16-5p has been shown to be decreased in AD (Gui et al., 2015), and MiR-16-5p targets TXNIP to regulate LPS-induced oxidative stress and apoptosis in cardiomyocytes (Yu-Cheng et al., 2020). Mir-17-5p inhibition decreased TXNIP/NLRP3 inflammasome activation after neonatal hypoxic–ischemic brain injury in rats (Chen et al., 2018). Mir-17-5p and mir-26b-5p may involved in apoptosis and proinflammatory cytokines in the pathogenesis of AD (Nguyen and Kim, 2022). Mir-16-5p is upregulated by amyloid *β* deposition in AD models and induces neuronal cell apoptosis through direct targeting and suppression of BCL-2 (Kim et al., 2020). The analysis of DEGs-TFs and DEGs-miRNA predicted that neuroinflammation mediates the progression of nHIBD to AD.

### 3.5 Identification of the drug candidates

To identify drug candidates that may affect the progression of nHIBD to AD, we studied protein-drug interactions, the analysis of which is important to understand the essential properties of sensitive receptors (Mahmud et al., 2021). The analysis of protein-drug interactions explains the drug-hub-protein interactions. Figure 6C shows the relationship between the drug UBIQUINONE-2 and FAMOXADONE and the protein CYC1 and UQCRFS1, the drug 17-Dmag and the protein NOS3, the drug Minocycline and the protein MAPKAPK2, and the drug Nedocromil and the protein HSP90AA1. Among the above-mentioned drugs, UBIQUINONE-2 is associated with cancer, FAMOXADONE is an antibacterial agent, 17-Dmag is an anti-tumor agent, and Minocycline has antibacterial and anti-inflammatory properties. It has been shown that Minocycline is potential in the treatment of AD and nHIBD for its anti-inflammatory effect, and the predicted receptor MAPKAPK2 is involved in inflammatory response by regulating tumor necrosis factor (TNF) and IL6 production post-transcriptionally (Tollenaere et al., 2015), which suggests that inflammation is a key mechanism in the development of nHIBD to AD (Min et al., 2020). FAMOXADON will promote the occurrence of AD, is one of the risk factors for the occurrence of neurodegenerative diseases such as AD (Pearson et al., 2016). 17-Dmag is a HSP90 inhibitor, HSP90 is a ubiquitous molecular chaperone, found to have an important role in averting protein misfolding and aggregation through inhibition of apoptotic activity in neuro-inflammatory diseases (Alam et al., 2017). Overactivation of cyclin-dependent kinase 5 (Cdk5) by p25 leads to neuroinflammation that leads to neurodegeneration in Parkinson’s disease (PD) and AD, Minocycline ameliorating Alzheimer’s-like Pathology via Inhibiting Cdk5/p25 Signaling (Zhao et al., 2022). Minocycline has been used in basic research in AD and nHIBD, and its function is associated with the inhibition of neuroinflammation. There are not many studies on the use of other drugs for AD and nHIBD, and whether they are related to neuroinflammation needs to be further verified.

### 3.6 Identification of gene-disease associations

The hypothesis that various diseases can be interconnected is based on the possibility that they often have one or more DEGs in common. Disease-specific therapeutic interface technology graph reveals the link between genes and disease. According to the network analysis study of gene-disease relationships, mammary neoplasms, neoplasm metastasis, schizophrenia, and brain ischemia diseases are the most relevant to the hub proteins we identified (Figure 6D). These are not only highly related to neurological diseases but also to inflammation. Loss of TP53 triggers WNT-dependent systemic inflammation to drive mammary neoplasms (Wellenstein et al., 2019). Perinatal inflammation promoted schizophrenia (Depino, 2018). Inflammation is increasingly being recognized as contributing negatively to brain ischemia diseases (Tang et al., 2012). We found that inflammation plays an important role in diseases associated with DEGs, which indirectly indicates that the common DEGs of nHIBD and AD and inflammation are closely related, neuroinflammation may play a vital role in the progression of nHIBD to AD.

### 3.7 Gene-inflammation and protein-inflammation association analysis

While retrieving the UniProt Knowledgebase we found that the DEG AREL1 (Apoptosis-resistant E3 ubiquitin protein ligase 1) and the hub proteins AKT1 and MAPK14 are related to inflammatory response. AREL1 is a DEG whose product is located in the cytosol. It modulates pulmonary inflammation by targeting SOCS2 for ubiquitination and subsequent degradation by the proteasome (Lear et al., 2019) and relates to regulation of neuroinflammatory response according to the GO annotations (GO: 0150077). AKT has an important role in the regulation of NF-kappa-B-dependent gene transcription and positively regulates the activity of CREB1 (cyclic AMP -response element binding protein; Du and Montminy, 1998). The overactivation of PI3K-AKT signaling pathway is caused by AKT1 gene amplification or activating mutations. Interestingly, inhibition of the mGluR5/PI3K-AKT pathway alleviates Alzheimer’s disease-like pathology through the activation of autophagy in 5 × FAD mice (Chen et al., 2023b). MAPK14 is one of the four p38 MAPKs which play an important role in the cascades of cellular responses evoked by extracellular stimuli such as pro-inflammatory cytokines or physical stress leading to direct activation of transcription factors. The promoters of several genes involved in the inflammatory response, such as IL6, IL8 and IL12B, display a p38 MAPK-dependent enrichment of histone H3 phosphorylation on ‘Ser-10’ (H3S10ph) in LPS-stimulated myeloid cells (Reinhardt et al., 2010). Activated MAPK14 protein can also activate downstream protein kinases and transcription factors, to upregulate the expression of inflammatory factors such as TNF-*α*, IL-2, and IL-4 to mediate apoptotic cell death (Zarubin and Han, 2005).

### 3.8 Behavioral analysis of nHIBD mice

**Figure 7:**
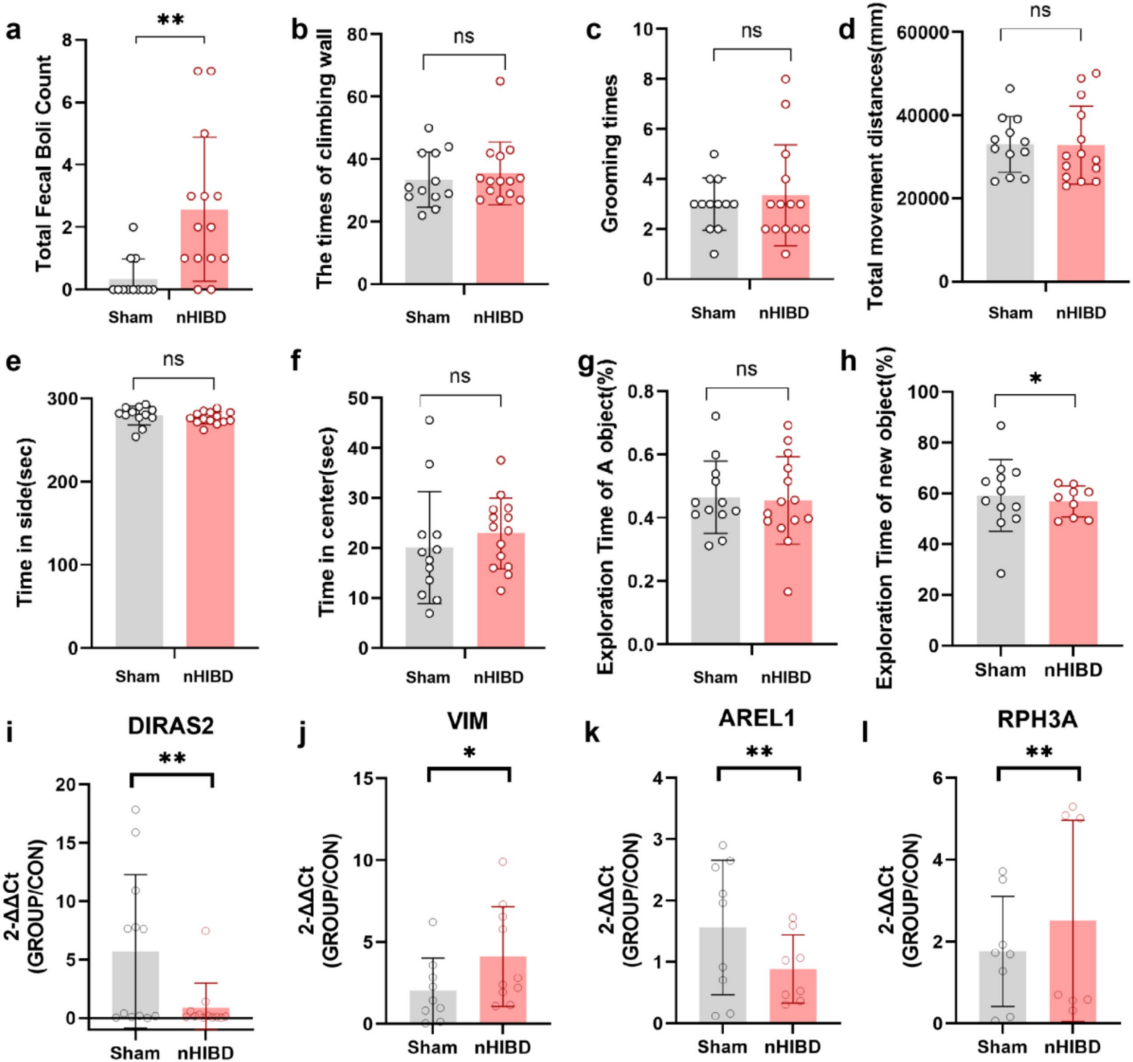
The results of the Open Field and Novel Object Recognition test and the expression levels of four DEGs in cortex tissues detected by RT-qPCR. **(A-F)**, Open Filed test. **(A)** The number of fecal boli. **(B)** The times of climbing wall. **(C)** The times of grooming. **(D)** Total movement distances. **(E)** Time spent in side. **(F)** Time spent in center. **(G,H)** Novel Object Recognition. **(G)** The exporation time of A object in the first day. **(H)** The exporation time of new object in the next day. **(I–L)** The results of qPCR tests on RNA. **(I)** The expression levels of DIRAS2 in cortex tissues detected by RT-qPCR. **(J)** The expression levels of VIM in cortex tissues detected by RT-qPCR. **(K)** The expression levels of AREL1 in cortex tissues detected by RT-qPCR. **(L)** The expression levels of RPH3A in cortex tissues detected by RT-qPCR. **p < 0.01. *p < 0.05. (*n* equal or greater than 8).

Compared with the sham group, the nHIBD group showed anxiety behavior and unaffected motor function (Figures 7A–D). For novel object recognition, the nHIBD mouse explored novel objects for less time than the sham group, and the time of exploring novel objects were statistically significant (*p* < 0.05). These data indicate that the nHIBD mice developed some anxiety and cognitive impairments (Figures 7E–H). Previous results from behavioral experiments showed that 2 months after modeling mice developed anxiety, and inflammation is one of the causes of anxiety (Zhuang et al., 2022). Moreover, studies have indicated that elderly people with anxiety disorders are more likely to have cognitive dysfunction, which is one of the characteristics of AD (Sun et al., 2023). Herein, the open field and novel object recognition results revealed anxiety and cognitive impairments in nHIBD mice, respectively. Anxiety has been shown to be one of the factors contributing to cognitive dysfunction, suggesting that the inflammation that occurs after nHIBD might trigger anxiety and cognitive dysfunction. Therefore, this may be an important mechanism that promotes the occurrence of AD. It has been proved that SD rats after nHIBD developed cognitive impairment 1 month after modeling by Morris water maze test, and our open field and new object recognition experiments are further validated by this experiment (Chen et al., 2024).

### 3.9 qPCR validation

Through GO and KEGG analysis, we found that these common DEGs are closely related to inflammatory function. Through PPI analysis, we found that DEGs and hub proteins are closely related to AD, MAPK signaling pathway, p53 signaling pathway, PI3K-Akt signaling pathway, and the latter three pathways are mainly involved in the process of inflammation regulation. Through the analysis of DEGs-TFs, DEGs-miRNA, DEGs-drugs, DEGs-diseases, we found that the related TFs, miRNA, drugs and diseases were also associated with inflammation. To demonstrate the accuracy of our genetic predictions, we validated them by performing common DEGs associated with inflammation. By querying, AREL1, VIM, DIRAS2, RPH3A were chosen for qPCR experiments to confirm this prediction. From the GO and KEGG analyses, we know that AREL1 is a gene associated with inflammation, AREL1 can limit inflammation by shrinking the cellular pool of pro-IL-1β (Mishra et al., 2023). VIM is a disease-associated astrocyte gene identified in one AD mouse model (Habib et al., 2020), it has been reported that infecting mouse brain with EV71-induced VIM gene-mediated NLRP3 inflammasome activation, leads to the release of IL-1β and caspase-1, which may be one of the causes of inflammation and neuronal injury in the CNS (Xiao et al., 2018). DIRAS2 involved in the progress of TBEV-infected neurons and astrocytes was revealed that DIRAS2 may be associated with inflammation (Selinger et al., 2022). RPH3A, LCN2, S100A8, S100A9 et al., belonging to pathways related to vasculature development, hypoxia, epithelial cell apoptosis, epithelial mesenchymal transition, and inflammation, support the pachychoroid phenotype and highlight downstream molecular targets (Leclercq et al., 2023). Interestingly, the expression of Diras2 and Arel1 in the sham group was higher than that of the nHIBD group, whereas the expression of Vim in the sham group was lower than that in the nHIBD group. These results are consistent with our gene expression results selected from the GEO database (Figures 7I–L).

## 4 Discussion

The GEO database was used to download and analyze two gene expression datasets, GSE23317 and GSE203206, and identified the DEGs common to nHIBD and AD. Using genetic data from nHIBD mice and AD patients, we performed bioinformatics analysis to characterize genes that are dysregulated in both diseases which could potentially serve as potential therapeutic candidates or diagnostic biomarkers. The 12 shared DEGs including *HSPB1, VIM, MVD, TUBB4A, AACS, ANXA6, DIRAS2, RPH3A, CEND1, KALM, THOP1, AREL1*, previous studies have shown that these DEGs are highly correlated with nHIBD and AD. *HSPB1* as promising targets involved in the pathological mechanisms of neurodegenerative diseases, is upregulated in AD (Fan et al., 2023). *HSPB1* overexpression improves hypoxic–ischemic brain damage by attenuating ferroptosis in rats through promoting G6PD expression (Dai and Hu, 2022). MiR-7a-2-3p plays a crucial role in the hypoxic–ischemic injury, and is associated with regulation of *VIM*, *VIM* is upregulated in nHIBD (Zhang et al., 2019). *TUBB4A* is correlated with tau inclusion formation at both transcriptomic and proteomic levels interact directly with or regulate tau, is downregulated in AD (Ficulle et al., 2022). In the nHIBD group, *TWS119* is found to be downregulated. Compared to the nHIBD group without *TWS119* treatment, treatment with *TWS119* up-regulated the expression of *CEND1* and promoted neuronal differentiation and cortical development, as evidenced by the expression of NeuN and TBR1 (Gao et al., 2024). *CEND1* is a neuronal specific protein and locates in the presynaptic mitochondria. Depletion of *CEND1* leads to increased mitochondrial fission mediated by upregulation of dynamin related protein 1 (Drp1), resulting in abnormal mitochondrial functions. *CEND1* deficiency leads to cognitive impairments in mice. Overexpression of CEND1 in the hippocampus of 5 × FAD mice rescued cognitive deficits (Xie et al., 2022). *RPH3A* loss correlated with dementia severity, cholinergic deafferentation, and increased *β*-amyloid (Aβ) concentrations (Tan et al., 2014). *THOP1* correlated with CSF total tau (t-tau), phosphorylated tau (p-tau), and Aβ40 (Rho >0.540) but not Aβ42 (Hok-A-Hin et al., 2023). The protein expressions of the DEGs mentioned above are consistent with our predictions.

The GO model provides a comprehensive theoretical framework for describing gene functions and their interactions within the field of gene expression, through GO and KEGG analysis, these DEGs were highly correlated with inflammation, this finding also provides the necessary preparation for the subsequent analysis of inflammation. It is progressively developed by gathering scientific knowledge about gene function and regulation, depending on the various GO categories and linguistic connections across categories (Rahman et al., 2021). The enrichment technology is used to explore and annotate three parts of the gene GO database (biological, cell, and molecule) for common genes. The biological component of the enrichment analysis GO includes biological processes like the regulation of the cellular process, cell-substrate adherent junction, cell-substrate junction, focal adhesion, neuron projection, and extracellular exosome, GTP binding, small molecule binding, purine ribonucleoside triphosphate binding, purine ribonucleotide binding, and purine nucleotide binding. The cell part refers to the cellular form in which genes control their activity, whereas the molecular concept refers to the molecular activity of the result of the gene expression. Pathway analysis is a new strategy that explores and reveals how biologically or molecularly complex diseases are related. This pathway is the best way to achieve organismal responses caused by internal changes in Signaling by Rho GTPases, Miro GTPases, and RHOBTB3, Signal Transduction, Adaptive Immune System, and Immune System (Podder et al., 2020). We found the common DEGs are involved in GO analysis and KEGG pathway studies have revealed many mechanisms in neurodegenerative diseases. The analysis showed that these DEGs associated with immune system and adaptive immune system. In addition, this procedure has been successful in identifying other major proteins or hub proteins that are shared between nHIBD and AD. It presents many new avenues of research, such as the possibility of being used for preventive therapy.

PPI experiments are usually performed to reach basic disease-related signaling molecules and pathways that may amplify the disease aspects of model systems (Rahman et al., 2020). Therefore, we performed PPI studies to identify key hubs. Interestingly, 11 hub proteins including AKT 1, TP 53, CYCS, HSPB 1, CASP 9, SNAP25, MDM 2, MAPK14, MAP3K5, DAXX, and HSP90AA1 were identified. In the GO and KEGG analyses, we found that inflammation plays an important role in the common DEGs of nHIBD and AD, so we focused on how the hub protein plays a role through inflammation. These hub proteins are potentially important biomarkers. Looking at the treatment possibilities of the diseases under study, we built a submodule network to better understand the close connection and proximity of the hub proteins with the help of Cytohubba plug-ins. The hub protein is the protein most closely associated with DEGs, and by identifying the hub protein, we can predict proteins that may play a key role in nHIBD and AD. AKT1 involved in IGF-I neuroprotection is downregulated in nHIBD (Brywe et al., 2005), and AKT1 is identified as the most potent regulators of the functional interaction network formed by these immune processes in AD (El Idrissi et al., 2021). TP53-induced glycolysis and apoptosis regulator (TIGAR) can protect neurons after cerebral ischemia/reperfusion (Tan et al., 2021). The roles and activities of miR-34a in the brain are modulated by factors that control its expression (such as Tp53/73), as well as its downstream target genes (such as the sirtuins SIRT1 and SIRT6) and signaling pathways (such as the Notch pathway), and the Notch signaling pathway regulates macrophage polarization (Chua and Tang, 2019). CYCS is pyroptosis-related gene in AD (Xia et al., 2023). HSPB1 is associated with ferroptosis in nHIBD, ferroptosis trigger the innate immune system by releasing inflammation-related damage-related molecules, and immune cells elicit an inflammatory response by recognizing mechanisms of different patterns of cell death (Dai and Hu, 2022). HSPB1 Modulate amyloid-*β* protein precursor expression in AD (Conway et al., 2014). CASP9 is a protein related with apoptosis and angiogenesis, chronic inflammation is often accompanied by angiogenesis and apoptotic cells control inflammation by selectively releasing metabolic molecules (Kumral et al., 2013). CASP9 is involved in neuronal inflammation and apoptosis in AD (Fang et al., 2018). SNAP25 attenuates neuronal injury via reducing ferroptosis in acute ischemic stroke (Si et al., 2023). SNAP25 is one of biomarker of AD, ameliorates postoperative cognitive dysfunction by facilitating PINK1-dependent mitophagy and impeding caspase-3/GSDME-dependent pyroptosis (Wang et al., 2023). MDM2 is key protein in nicotinic acid supplementation which contributes to the amelioration of AD in mouse models (Wang et al., 2022). MAPK14 is associated with ferroptosis in AD, P38*α*-MAPK phosphorylates Snapin and reduces Snapin-mediated BACE1 transportation in APP-transgenic mice (Schnöder et al., 2021). MAP3K5 is associated with many signaling pathways, which include endoplasmic reticulum (ER) stress-mediated apoptosis, Aβ-induced neurotoxicity, tau protein phosphorylation, and insulin signal transduction in AD (Song et al., 2014). Estrogen protects against amyloid-β toxicity by estrogen receptor α-mediated inhibition of Daxx translocation (Mateos et al., 2012). HSP90AA1 is involved in pyroptosis in nHIBD, pyroptosis is an inflammatory death of cells (Hou et al., 2020). HSP90AA1 is one of immunogenic cell death-related genes involved in AD (Wang et al., 2024). We can find that these hub proteins are mainly associated with inflammatory processes, pyroptosis, ferroptosis, and apoptosis.

Previous studies have shown that inflammation may play an important role in these two diseases, and for further validation, we analyzed the role of inflammation in common DEGs-related TFs, miRNAs, diseases, and drugs. Through the search of the path on the KEGG website,^5^ we found that the screened DEGs and hub proteins were mainly concentrated in Alzheimer disease pathway, MAPK signaling pathway, p53 signaling pathway, PI3K-Akt signaling pathway (Figure 5C), the latter three are mainly involved in the regulation of inflammation. To classify the DEG of regulatory molecules, we examined transcription factors and miRNAs of important regulatory molecules. In terms of DEGs-TF interaction, we identified 3 transcription factors, STAT3, SP1, and NFkB. Previous studies have shown that mutations in STAT3 are associated with neurodegenerative diseases such as AD, whereas SP1 is associated with cardiovascular diseases, and NFkB is associated with hypoxic and ischemic disease (Zaghloul et al., 2020; Zhang et al., 2021; Zhang et al., 2018). Previous studies have suggested that advancements in bioinformatics approaches and high-throughput technologies for TF and miRNA target prediction, as well as the integration of multilevel networks, hold promises for the future of medical science even though there are challenges (Zhang et al., 2015). Nowadays, miRNA is becoming increasingly prominent as a biomarker for many complex diseases, including cancer. We here identified three major regulatory miRNAs including mir-17-5p, mir-26b-5p, and mir-16-5p in our study. Among them, microRNA-17-5p is a novel endothelial cell modulator and controls vascular reendothelialization and neointimal lesion formation, whereas MiR-26b-5p regulates the preadipocyte differentiation by targeting FGF21 in goats, and MiR-16-5p regulates postmenopausal osteoporosis by directly targeting VEGFA (Liu et al., 2023; Ma et al., 2021; Yu et al., 2020). Studies of gene-disease interaction networks have identified comorbidities associated with hub genes. The diseases about which we identified central genes that were the most in sync were Mammary Neoplasms, Neoplasm Metastasis, Schizophrenia, and Brain Ischemia diseases. Further, to search for new drugs targeting the hub proteins, we explored protein-drug interactions and our results revealed that CNF1010, 17-Dmag, and Nedocromil were related to the hub protein HSP90AA1, whereas Gambogic acid and Zinc were related to the key common proteins DEGs S100A8 and S100A9. These key genes were found by the gene-drug database and were associated with therapeutic anti-inflammatory drugs such as minocycline, indicating that inflammation plays an important role in the progression of nHIBD to AD. Although the association between these drugs and proteins has been established, more research is needed to determine whether treating patients with nHIBD with these drugs can decrease the likelihood of developing AD. Directly using drugs as variables to judge the curative effect of drugs on diseases is the most direct criterion, and it may also be the direction of follow-up research (Khanal et al., 2024a; Khanal et al., 2023; Khanal et al., 2024b). Finally, we analyzed the inflammation-associated DEGs and hub proteins in an attempt to find a common mechanism as the direct link between these two diseases and inflammation. As illustrated by open field and novel object recognition behavior tests, mice developed anxiety and cognitive dysfunction after nHIBD. This indicates that brain inflammation in nHIBD mice leads to anxiety, and anxiety plays an important role in the development of cognitive dysfunction which to a certain extent may develop into AD. Consistently, a previous study revealed that common neuropsychiatric symptoms such as irritability seen in AD patients are the consequence of brain inflammation rather than Aβ and tau pathologies (Schaffer Aguzzoli et al., 2023), and the qPCR results verified the DEGs.

## 5 Conclusion

**Figure 8:**
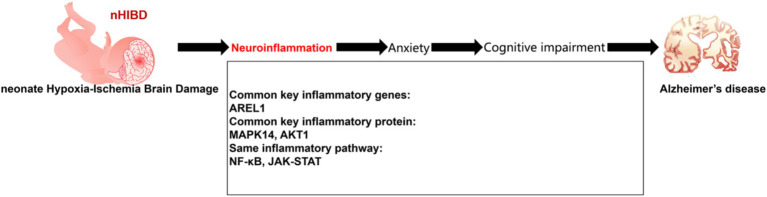
Proposed model of development of AD from nHIBD with neuroinflammation playing an important role.

Bioinformatics studies identified DEGs common to nHIBD and AD that were related to inflammation, indicating that inflammation plays an important role in the pathogenesis of nHIBD and AD. Data from behavioral experiments showed that nHIBD mice exhibit anxiety and cognitive impairments, and patients with anxiety disorders are more likely to develop AD. Therefore, we propose a mechanism for nHIBD progression to AD and offer a potential therapeutic approach (Figure 8).

The clinical enlightenment of this pathogenesis is that we can reduce inflammation by paying attention to the inflammation and anxiety of nHIBD patients, through relevant drug treatment, and intervening in the screened inflammation-related genes, and we can judge the clinical treatment effect by observing the anxiety of patients for a long time.

The sample size in some studies may be insufficient to describe all essential disease-related genes required to identify frequent DEGs. But the innovation of our research is that we connect early on-stage diseases with end-stage diseases in the hope of finding treatments during brain development. With the deepening of research, it is of great significance to clinical research.

## Data Availability

Publicly available datasets were analyzed in this study. This data can be found at: https://www.ncbi.nlm.nih.gov/geo/query/acc.cgi?acc=GSE203206 (GSE203206), https://www.ncbi.nlm.nih.gov/geo/query/acc.cgi?acc=GSE23317 (GSE23317).

## Footnotes

^1^ http://www.ncbi.nlm.nih.gov/geo

^2^STRING; http://string-db.org

^3^version 3.0, https://www.networkanalyst.ca/

^4^ https://www.uniprot.org/

^5^ https://www.genome.jp/

## References

[ref1] Alam Q. Alam M. Z. Sait K. H. W. Anfinan N. Noorwali A. W. Kamal M. A.. (2017). Translational shift of HSP90 as a novel therapeutic target from cancer to neurodegenerative disorders: an emerging trend in the cure of Alzheimer’s and Parkinson’s diseases. Curr. Drug Metab. 18, 868–876. doi: 10.2174/1389200218666170728115606, PMID: 28758577

[ref2] Alzheimer’s Association (2017). 2017 Alzheimer's disease facts and figures. Alzheimers Dement. 13, 325–373. doi: 10.1016/j.jalz.2017.02.001

[ref3] Brywe K. G. Mallard C. Gustavsson M. Hedtjärn M. Leverin A. L. Wang X.. (2005). IGF-I neuroprotection in the immature brain after hypoxia-ischemia, involvement of Akt and GSK3beta? Eur. J. Neurosci. 21, 1489–1502. doi: 10.1111/j.1460-9568.2005.03982.x, PMID: 15845077

[ref4] Caldwell A. B. Anantharaman B. G. Ramachandran S. Nguyen P. Liu Q. Trinh I. (2022). Transcriptomic profiling of sporadic Alzheimer's disease patients. Mol. Brain 15:83. doi: 10.1186/s13041-022-00963-2, PMID: 36224601 PMC9559068

[ref5] Chandra A. Farrell C. Wilson H. Dervenoulas G. De Natale E. R. Politis M. (2021). Aquaporin-4 polymorphisms predict amyloid burden and clinical outcome in the Alzheimer's disease spectrum. Neurobiol. Aging 97, 1–9. doi: 10.1016/j.neurobiolaging.2020.06.007, PMID: 33068891

[ref6] Chen X. Chen A. Wei J. Huang Y. Deng J. Chen P.. (2024). Dexmedetomidine alleviates cognitive impairment by promoting hippocampal neurogenesis via BDNF/TrkB/CREB signaling pathway in hypoxic-ischemic neonatal rats. CNS Neurosci. Ther. 30:e14486. doi: 10.1111/cns.14486, PMID: 37830170 PMC10805444

[ref7] Chen D. Dixon B. J. Doycheva D. M. Li B. Zhang Y. Hu Q.. (2018). IRE1α inhibition decreased TXNIP/NLRP3 inflammasome activation through miR-17-5p after neonatal hypoxic-ischemic brain injury in rats. J. Neuroinflammation 15:32. doi: 10.1186/s12974-018-1077-9, PMID: 29394934 PMC5797348

[ref8] Chen Y. Li X. Xiong Q. Du Y. Luo M. Yi L.. (2023b). Inhibiting NLRP3 inflammasome signaling pathway promotes neurological recovery following hypoxic-ischemic brain damage by increasing p97-mediated surface GluA1-containing AMPA receptors. J. Transl. Med. 21:567. doi: 10.1186/s12967-023-04452-5, PMID: 37620837 PMC10463885

[ref9] Chen Y. Zhang Y. Chen Q. Liu Y. Wei X. Wu M. (2023a). Inhibition of mGluR5/PI3K-AKT pathway alleviates Alzheimer's disease-like pathology through the activation of autophagy in 5XFAD mice. J. Alzheimers Dis. 91, 1197–1214. doi: 10.3233/JAD-221058, PMID: 36565127

[ref10] Chua C. E. L. Tang B. L. (2019). miR-34a in neurophysiology and neuropathology. J. Mol. Neurosci. 67, 235–246. doi: 10.1007/s12031-018-1231-y, PMID: 30488149

[ref12] Conway M. Nafar F. Straka T. Mearow K. (2014). Modulation of amyloid-β protein precursor expression by HspB1. J. Alzheimer’s Dis. 42, 435–450. doi: 10.3233/JAD-140348, PMID: 24898650

[ref13] Dai Y. Hu L. (2022). HSPB1 overexpression improves hypoxic-ischemic brain damage by attenuating ferroptosis in rats through promoting G6PD expression. J. Neurophysiol. 128, 1507–1517. doi: 10.1152/jn.00306.2022, PMID: 36321738

[ref14] Depino A. M. (2018). Perinatal inflammation and adult psychopathology: from preclinical models to humans. Semin. Cell Dev. Biol. 77, 104–114. doi: 10.1016/j.semcdb.2017.09.010, PMID: 28890420

[ref15] Douglas-Escobar M. Weiss M. D. (2015). Hypoxic-ischemic encephalopathy: a review for the clinician. JAMA Pediatr. 169, 397–403. doi: 10.1001/jamapediatrics.2014.3269 25685948

[ref16] Du K. Montminy M. (1998). CREB is a regulatory target for the protein kinase Akt/PKB. J. Biol. Chem. 273, 32377–32379. doi: 10.1074/jbc.273.49.32377, PMID: 9829964

[ref17] Edgar R. Domrachev M. Lash A. E. (2002). Gene expression omnibus: NCBI gene expression and hybridization array data repository. Nucleic Acids Res. 30, 207–210. doi: 10.1093/nar/30.1.207, PMID: 11752295 PMC99122

[ref18] El Idrissi F. Gressier B. Devos D. Belarbi K. (2021). A computational exploration of the molecular network associated to neuroinflammation in Alzheimer's disease. Front. Pharmacol. 12:630003. doi: 10.3389/fphar.2021.630003, PMID: 34335238 PMC8319636

[ref19] Fan L. Y. Yang J. Liu R. Y. Kong Y. Guo G. Y. Xu Y. M. (2023). Integrating single-nucleus sequence profiling to reveal the transcriptional dynamics of Alzheimer's disease, Parkinson's disease, and multiple sclerosis. J. Transl. Med. 21:649. doi: 10.1186/s12967-023-04516-6, PMID: 37735671 PMC10515258

[ref20] Fang X. X. Sun G. L. Zhou Y. Qiu Y. H. Peng Y. P. (2018). TGF-β1 protection against Aβ1-42-induced hippocampal neuronal inflammation and apoptosis by TβR-I. Neuroreport 29, 141–146. doi: 10.1097/WNR.0000000000000940, PMID: 29200096

[ref21] Faraco G. Hochrainer K. Segarra S. G. Schaeffer S. Santisteban M. M. Menon A.. (2019). Dietary salt promotes cognitive impairment through tau phosphorylation. Nature 574, 686–690. doi: 10.1038/s41586-019-1688-z, PMID: 31645758 PMC7380655

[ref22] Ficulle E. Kananathan S. Airey D. Gharbi S. I. Humphryes-Kirilov N. Scherschel J.. (2022). A human tau seeded neuronal cell model recapitulates molecular responses associated with Alzheimer's disease. Sci. Rep. 12:2673. doi: 10.1038/s41598-022-06411-4, PMID: 35177665 PMC8854741

[ref23] Gao L. Gao S. Shan H. Wu Y. Zhou Q. (2024). GSK-3β inhibitor TWS119 promotes neuronal differentiation after hypoxic-ischemic brain damage in neonatal rats. Neuroreport 35, 200–207. doi: 10.1097/WNR.0000000000002006, PMID: 38305107 PMC10833190

[ref24] Gui Y. Liu H. Zhang L. Lv W. Hu X. (2015). Altered microRNA profiles in cerebrospinal fluid exosome in Parkinson disease and Alzheimer disease. Oncotarget 6, 37043–37053. doi: 10.18632/oncotarget.6158, PMID: 26497684 PMC4741914

[ref25] Habib N. McCabe C. Medina S. Varshavsky M. Kitsberg D. Dvir-Szternfeld R.. (2020). Disease-associated astrocytes in Alzheimer's disease and aging. Nat. Neurosci. 23, 701–706. doi: 10.1038/s41593-020-0624-8, PMID: 32341542 PMC9262034

[ref26] Heneka M. T. Kummer M. P. Stutz A. Delekate A. Schwartz S. Vieira-Saecker A.. (2013). NLRP3 is activated in Alzheimer's disease and contributes to pathology in APP/PS1 mice. Nature 493, 674–678. doi: 10.1038/nature11729, PMID: 23254930 PMC3812809

[ref27] Hok-A-Hin Y. S. Bolsewig K. Ruiters D. N. Lleó A. Alcolea D. Lemstra A. W.. (2023). Thimet oligopeptidase as a potential CSF biomarker for Alzheimer's disease: A cross-platform validation study. Alzheimers Dement. 15:e12456. doi: 10.1002/dad2.12456, PMID: 37502019 PMC10369371

[ref28] Hou X. Yuan Z. Wang X. Cheng R. Zhou X. Qiu J. (2020). Peptidome analysis of cerebrospinal fluid in neonates with hypoxic-ischemic brain damage. Mol. Brain 13:133. doi: 10.1186/s13041-020-00671-9, PMID: 33008433 PMC7531121

[ref29] Huang F. Wang M. Liu R. Wang J. Z. Schadt E. Haroutunian V.. (2019). CDT2-controlled cell cycle reentry regulates the pathogenesis of Alzheimer's disease. Alzheimers Dement. 15, 217–231. doi: 10.1016/j.jalz.2018.08.013, PMID: 30321504 PMC6758558

[ref30] Huang J. Zuber V. Matthews P. M. Elliott P. Tzoulaki J. Dehghan A. (2020). Sleep, major depressive disorder, and Alzheimer disease: A Mendelian randomization study. Neurology 95, e1963–e1970. doi: 10.1212/WNL.0000000000010463, PMID: 32817390 PMC7682841

[ref31] Khanal P. Patil V. S. Bhattacharya K. Patil B. M. (2024b). Multifaceted targets of cannabidiol in epilepsy: modulating glutamate signaling and beyond. Comput. Biol. Med. 179:108898. doi: 10.1016/j.compbiomed.2024.108898, PMID: 39047503

[ref32] Khanal P. Patil V. S. Bhattacharya K. Shrivastava A. K. Bhandare V. V. (2024a). Exploring the globoid cell leukodystrophy protein network and therapeutic interventions. Sci. Rep. 14:18067. doi: 10.1038/s41598-024-66437-8, PMID: 39103379 PMC11300594

[ref33] Khanal P. Patil V. S. Patil B. M. Bhattacharya K. Shrivastava A. K. Chaudhary R. K.. (2023). The marijuana-schizophrenia multifaceted nexus: connections and conundrums towards neurophysiology. Comput. Biol. Chem. 107:107957. doi: 10.1016/j.compbiolchem.2023.107957, PMID: 37729848

[ref34] Kim Y. J. Kim S. H. Park Y. Park J. Lee J. H. Kim B. C.. (2020). miR-16-5p is upregulated by amyloid β deposition in Alzheimer's disease models and induces neuronal cell apoptosis through direct targeting and suppression of BCL-2. Exp. Gerontol. 136:110954. doi: 10.1016/j.exger.2020.110954, PMID: 32320719

[ref35] Kraeuter A. K. Guest P. C. Sarnyai Z. (2019). The open field test for measuring locomotor activity and anxiety-like behavior. Methods Mol. Biol. 1916, 99–103. doi: 10.1007/978-1-4939-8994-2_9 30535687

[ref36] Kumral A. Tuzun F. Tugyan K. Ozbal S. Yılmaz O. Yesilirmak C. D.. (2013). Role of epigenetic regulatory mechanisms in neonatal hypoxic-ischemic brain injury. Early Hum. Dev. 89, 165–173. doi: 10.1016/j.earlhumdev.2012.09.016, PMID: 23046993

[ref37] Kurinczuk J. J. White-Koning M. Badawi N. (2010). Epidemiology of neonatal encephalopathy and hypoxic-ischaemic encephalopathy. Early Hum. Dev. 86, 329–338. doi: 10.1016/j.earlhumdev.2010.05.010 20554402

[ref38] Lear T. B. McKelvey A. C. Evankovich J. W. Rajbhandari S. Coon T. A. Dunn S. R.. (2019). KIAA0317 regulates pulmonary inflammation through SOCS2 degradation. JCI Insight 4:e129110. doi: 10.1172/jci.insight.129110, PMID: 31578312 PMC6795399

[ref39] Leclercq B. Weiner A. Zola M. Mejlacowicz D. Lassiaz P. Jonet L.. (2023). The choroidal nervous system: a link between mineralocorticoid receptor and pachychoroid. Acta Neuropathol. 146, 747–766. doi: 10.1007/s00401-023-02628-3, PMID: 37682293 PMC10564818

[ref40] Li T. T. Xin D. Q. Ke H. F. Chu X. L. Zhao Y. J. Yue S. W.. (2022). L-cysteine attenuates osteopontin-mediated neuroinflammation following hypoxia-ischemia insult in neonatal mice by inducing S-sulfhydration of Stat3. Acta Pharmacol. Sin. 43, 1658–1669. doi: 10.1038/s41401-021-00794-2, PMID: 34737419 PMC9253102

[ref41] Liu X. Chen J. Liu G. Zhang B. Jin X. Wang Y. (2023). MicroRNA-17-5p, a novel endothelial cell modulator, controls vascular re-endothelialization and neointimal lesion formation. Vascular 31, 392–401. doi: 10.1177/17085381211067672, PMID: 34958294

[ref42] Lukiw W. J. (2022). NF-kB (p50/p65)-mediated pro-inflammatory microRNA (miRNA) signaling in Alzheimer's disease (AD). Front. Mol. Neurosci. 15:943492. doi: 10.3389/fnmol.2022.943492, PMID: 35836546 PMC9274251

[ref43] Ma J. Lin Y. Zhu J. Huang K. Wang Y. (2021). MiR-26b-5p regulates the preadipocyte differentiation by targeting FGF21 in goats. In vitro cellular & developmental biology. Animal 57, 257–263. doi: 10.1007/s11626-020-00493-y, PMID: 33511524

[ref44] Mahmud S. M. H. Chen W. Liu Y. Awal M. A. Ahmed K. Rahman M. H. (2021). PreDTIs: prediction of drug-target interactions based on multiple feature information using gradient boosting framework with data balancing and feature selection techniques. Brief. Bioinform. 22:bbab046. doi: 10.1093/bib/bbab046, PMID: 33709119 PMC7989622

[ref45] Masseroli M. (2019). *Biological and medical ontologies: GO and GOA*. Encyclopedia of Bioinformatics and Computational Biology.

[ref46] Mateos L. Persson T. Katoozi S. Gil-Bea F. J. Cedazo-Minguez A. (2012). Estrogen protects against amyloid-β toxicity by estrogen receptor α-mediated inhibition of Daxx translocation. Neurosci. Lett. 506, 245–250. doi: 10.1016/j.neulet.2011.11.016, PMID: 22119000

[ref47] Mi Y. Qi G. Vitali F. Shang Y. Raikes A. C. Wang T.. (2023). Loss of fatty acid degradation by astrocytic mitochondria triggers neuroinflammation and neurodegeneration. Nat. Metab. 5, 445–465. doi: 10.1038/s42255-023-00756-4, PMID: 36959514 PMC10202034

[ref48] Min Y. J. Ling E. A. Li F. (2020). Immunomodulatory mechanism and potential therapies for perinatal hypoxic-ischemic brain damage. Front. Pharmacol. 11:580428. doi: 10.3389/fphar.2020.580428, PMID: 33536907 PMC7849181

[ref49] Mishra V. Crespo-Puig A. McCarthy C. Masonou T. Glegola-Madejska I. Dejoux A.. (2023). IL-1β turnover by the UBE2L3 ubiquitin conjugating enzyme and HECT E3 ligases limits inflammation. Nat. Commun. 14:4385. doi: 10.1038/s41467-023-40054-x 37474493 PMC10359330

[ref50] Nguyen H. D. Kim M. S. (2022). Exposure to a mixture of heavy metals induces cognitive impairment: genes and microRNAs involved. Toxicology 471:153164. doi: 10.1016/j.tox.2022.153164, PMID: 35346790

[ref51] Nong W. Bao C. Chen Y. Wei Z. (2022). miR-212-3p attenuates neuroinflammation of rats with Alzheimer's disease via regulating the SP1/BACE1/NLRP3/Caspase-1 signaling pathway. Bosn. J. Basic Med. Sci. 22, 540–552. doi: 10.17305/bjbms.2021.6723, PMID: 35150479 PMC9392983

[ref52] Pearson B. L. Simon J. M. McCoy E. S. Salazar G. Fragola G. Zylka M. J. (2016). Identification of chemicals that mimic transcriptional changes associated with autism, brain aging and neurodegeneration. Nat. Commun. 7:11173. doi: 10.1038/ncomms11173, PMID: 27029645 PMC4821887

[ref53] Piñero J. Bravo À. Queralt-Rosinach N. Gutiérrez-Sacristán A. Deu-Pons J. Centeno E. (2017). DisGeNET: a comprehensive platform integrating information on human disease-associated genes and variants. Nucleic Acids Res. 45, D833–D839. doi: 10.1093/nar/gkw943, PMID: 27924018 PMC5210640

[ref54] Podder N. K. Rana H. K. Azam S. Rana S. Akhtar R. Rahman R. (2020). A system biological approach to investigate the genetic profiling and comorbidities of type 2 diabetes. Gene Rep. 21:100830. doi: 10.1016/j.genrep.2020.100830

[ref55] Rahman M. H. Peng S. Hu X. Chen C. Rahman M. R. Uddin S. (2020). A network-based bioinformatics approach to identify molecular biomarkers for type 2 diabetes that are linked to the progression of neurological diseases. Int. J. Environ. Res. Public Health 17:1035. doi: 10.3390/ijerph17031035, PMID: 32041280 PMC7037290

[ref56] Rahman M. H. Rana H. K. Peng S. Hu X. Chen C. Quinn J. M. W. (2021). Bioinformatics and machine learning methodologies to identify the effects of central nervous system disorders on glioblastoma progression. Brief. Bioinform. 22:bbaa365. doi: 10.1093/bib/bbaa365, PMID: 33406529

[ref57] Rain J. C. Selig L. De Reuse H. Battaglia V. Reverdy C. Simon S. (2001). The protein-protein interaction map of *Helicobacter pylori*. Nature 409, 211–215. doi: 10.1038/35051615 11196647

[ref58] Reinhardt H. C. Hasskamp P. Schmedding I. Morandell S. van Vugt M. A. Wang X.. (2010). DNA damage activates a spatially distinct late cytoplasmic cell-cycle checkpoint network controlled by MK2-mediated RNA stabilization. Mol. Cell 40, 34–49. doi: 10.1016/j.molcel.2010.09.018, PMID: 20932473 PMC3030122

[ref59] Rice J. E. Vannucci R. C. Brierley J. B. (1981). The influence of immaturity on hypoxic-ischemic brain damage in the rat. Ann. Neurol. 9, 131–141. doi: 10.1002/ana.410090206, PMID: 7235629

[ref60] Ritchie M. E. Phipson B. Wu D. Hu Y. Law C. W. Shi W.. (2015). Limma powers differential expression analyses for RNA-sequencing and microarray studies. Nucleic Acids Res. 43:e47. doi: 10.1093/nar/gkv007, PMID: 25605792 PMC4402510

[ref61] Ruganzu J. B. Peng X. He Y. Wu X. Zheng Q. Ding B. (2022). Downregulation of TREM2 expression exacerbates neuroinflammatory responses through TLR4-mediated MAPK signaling pathway in a transgenic mouse model of Alzheimer's disease. Mol. Immunol. 142, 22–36. doi: 10.1016/j.molimm.2021.12.018 34959070

[ref62] Sabatino J. J. Pröbstel A. K. Zamvil S. S. (2019). B cells in autoimmune and neurodegenerative central nervous system diseases. Nat. Rev. Neurosci. 20, 728–745. doi: 10.1038/s41583-019-0233-2 31712781

[ref63] Schaffer Aguzzoli C. Ferreira P. C. L. Povala G. Ferrari-Souza J. P. Bellaver B. Soares Katz C.. (2023). Neuropsychiatric symptoms and microglial activation in patients with Alzheimer disease. JAMA Netw. Open 6:e2345175. doi: 10.1001/jamanetworkopen.2023.45175, PMID: 38010651 PMC10682836

[ref64] Scheckel C. Drapeau E. Frias M. A. Park C. Y. Fak J. Zucker-Scharff I. (2016). Regulatory consequences of neuronal ELAV-like protein binding to coding and non-coding RNAs in human brain. eLife 5:e10421. doi: 10.7554/eLife.10421 26894958 PMC4798961

[ref65] Schnöder L. Tomic I. Schwindt L. Helm D. Rettel M. Schulz-Schaeffer W.. (2021). P38α-MAPK phosphorylates Snapin and reduces Snapin-mediated BACE1 transportation in APP-transgenic mice. FASEB J. 35:e21691. doi: 10.1096/fj.202100017R, PMID: 34118085

[ref66] Selinger M. Věchtová P. Tykalová H. Ošlejšková P. Rumlová M. Štěrba J.. (2022). Integrative RNA profiling of TBEV-infected neurons and astrocytes reveals potential pathogenic effectors. Comput. Struct. Biotechnol. J. 20, 2759–2777. doi: 10.1016/j.csbj.2022.05.052, PMID: 35685361 PMC9167876

[ref67] Si W. Sun B. Luo J. Li Z. Dou Y. Wang Q. (2023). Snap25 attenuates neuronal injury via reducing ferroptosis in acute ischemic stroke. Exp. Neurol. 367:114476. doi: 10.1016/j.expneurol.2023.114476 37393984

[ref68] Song J. Park K. A. Lee W. T. Lee J. E. (2014). Apoptosis signal regulating kinase 1 (ASK1): potential as a therapeutic target for Alzheimer's disease. Int. J. Mol. Sci. 15, 2119–2129. doi: 10.3390/ijms15022119, PMID: 24481061 PMC3958840

[ref69] Sun X. Hu Y. Zhou H. Wang S. Zhou C. Lin L.. (2022). Inhibition of progesterone receptor membrane component-1 exacerbates neonatal hypoxic-ischemic cerebral damage in male mice. Exp. Neurol. 347:113893. doi: 10.1016/j.expneurol.2021.113893, PMID: 34653511

[ref70] Sun L. Li W. Qiu Q. Hu Y. Yang Z. Xiao S.. (2023). Anxiety adds the risk of cognitive progression and is associated with axon/synapse degeneration among cognitively unimpaired older adults. EBioMedicine 94:104703. doi: 10.1016/j.ebiom.2023.104703, PMID: 37429081 PMC10435838

[ref71] Szklarczyk D. Gable A. L. Lyon D. Junge A. Wyder S. Huerta-Cepas J. (2019). STRING v11: protein-protein association networks with increased coverage, supporting functional discovery in genome-wide experimental datasets. Nucleic Acids Res. 47, D607–D613. doi: 10.1093/nar/gky1131, PMID: 30476243 PMC6323986

[ref72] Tan L. L. Jiang X. L. Xu L. X. Li G. Feng C. X. Ding X.. (2021). TP53-induced glycolysis and apoptosis regulator alleviates hypoxia/ischemia-induced microglial pyroptosis and ischemic brain damage. Neural Regen. Res. 16, 1037–1043. doi: 10.4103/1673-5374.300453, PMID: 33269748 PMC8224121

[ref73] Tan M. G. Lee C. Lee J. H. Francis P. T. Williams R. J. Ramírez M. J.. (2014). Decreased rabphilin 3A immunoreactivity in Alzheimer's disease is associated with Aβ burden. Neurochem. Int. 64, 29–36. doi: 10.1016/j.neuint.2013.10.013, PMID: 24200817

[ref74] Tang X. N. Cairns B. Kim J. Y. Yenari M. A. (2012). NADPH oxidase in stroke and cerebrovascular disease. Neurol. Res. 34, 338–345. doi: 10.1179/1743132812Y.0000000021, PMID: 22643077 PMC3645888

[ref75] Tarkowska A. (2021). “Hypoxic-ischemic brain injury after perinatal asphyxia as a possible factor in the pathology of Alzheimer’s disease” in Cerebral Ischemia. ed. Pluta R. (Queensland: Exon Publications). 34905310

[ref76] Tollenaere M. A. X. Villumsen B. H. Blasius M. Nielsen J. C. Wagner S. A. Bartek J.. (2015). p38- and MK2-dependent signalling promotes stress-induced centriolar satellite remodelling via 14-3-3-dependent sequestration of CEP131/AZI1. Nat. Commun. 6:10075. doi: 10.1038/ncomms10075, PMID: 26616734 PMC4674683

[ref11] UniProt Consortium (2021). UniProt: the universal protein knowledgebase in 2021. Nucleic Acids Res. 49, D480–D489. doi: 10.1093/nar/gkaa1100, PMID: 33237286 PMC7778908

[ref77] Wang R. Du Y. Shao W. Wang J. Liu X. Xu X.. (2024). Identification of immunogenic cell death-related genes involved in Alzheimer's disease. Sci. Rep. 14:3786. doi: 10.1038/s41598-024-54357-6, PMID: 38360834 PMC10869701

[ref78] Wang W. Gao W. Zhang L. Xia Z. Zhao B. (2023). SNAP25 ameliorates postoperative cognitive dysfunction by facilitating PINK1-dependent mitophagy and impeding caspase-3/GSDME-dependent pyroptosis. Exp. Neurol. 367:114463. doi: 10.1016/j.expneurol.2023.114463, PMID: 37295545

[ref79] Wang M. Tang G. Zhou C. Guo H. Hu Z. Hu Q. (2023). Revisiting the intersection of microglial activation and neuroinflammation in Alzheimer's disease from the perspective of ferroptosis. Chem. Biol. Interact. 375:110387. doi: 10.1016/j.cbi.2023.110387, PMID: 36758888

[ref80] Wang Z. Zou Z. Li Q. (2022). Nicotinic acid supplementation contributes to the amelioration of Alzheimer's disease in mouse models. Ann. Transl. Med. 10:1049. doi: 10.21037/atm-22-1148, PMID: 36330413 PMC9622504

[ref81] Wellenstein M. D. Coffelt S. B. Duits D. E. M. van Miltenburg M. H. Slagter M. de Rink I.. (2019). Loss of p53 triggers WNT-dependent systemic inflammation to drive breast cancer metastasis. Nature 572, 538–542. doi: 10.1038/s41586-019-1450-6, PMID: 31367040 PMC6707815

[ref82] Wu Q. Chen W. Sinha B. Tu Y. Manning S. Thomas N. (2015). Neuroprotective agents for neonatal hypoxic-ischemic brain injury. Drug Discov. Today 20, 1372–1381. doi: 10.1016/j.drudis.2015.09.001 26360053

[ref83] Xia J. Gill E. E. Hancock R. E. (2015). Network analyst for statistical, visual and network-based meta-analysis of gene expression data. Nat. Protoc. 10, 823–844. doi: 10.1038/nprot.2015.052, PMID: 25950236

[ref84] Xia P. Ma H. Chen J. Liu Y. Cui X. Wang C.. (2023). Differential expression of pyroptosis-related genes in the hippocampus of patients with Alzheimer's disease. BMC Med. Genet. 16:56. doi: 10.1186/s12920-023-01479-x, PMID: 36918839 PMC10012531

[ref85] Xiao H. S. Xie Q. Zhong J. Y. Gerald Rukundo B. He X. L. Qu Y. L.. (2018). Effect of vimentin on activation of NLRP3 inflammasome in the brain of mice with EV71 infection. Nan Fang Yi Ke Da Xue Xue Bao 38, 704–710. doi: 10.3969/j.issn.1673-4254.2018.06.10, PMID: 29997093 PMC6765721

[ref86] Xie W. Guo D. Li J. Yue L. Kang Q. Chen G.. (2022). CEND1 deficiency induces mitochondrial dysfunction and cognitive impairment in Alzheimer's disease. Cell Death Differ. 29, 2417–2428. doi: 10.1038/s41418-022-01027-7, PMID: 35732922 PMC9751129

[ref87] Yang L. Zhao H. Cui H. (2020). Treatment and new progress of neonatal hypoxic-ischemic brain damage. Histol. Histopathol. 35, 929–936. doi: 10.14670/HH-18-214, PMID: 32167570

[ref88] Yu L. Yi J. Ye G. Zheng Y. Song Z. Yang Y. (2012). Effects of curcumin on levels of nitric oxide synthase and AQP-4 in a rat model of hypoxia-ischemic brain damage. Brain Res. 1475, 88–95. doi: 10.1016/j.brainres.2012.07.055, PMID: 22902770

[ref89] Yu T. You X. Zhou H. He W. Li Z. Li B. (2020). MiR-16-5p regulates postmenopausal osteoporosis by directly targeting VEGFA. Aging 12, 9500–9514. doi: 10.18632/aging.103223, PMID: 32427128 PMC7288956

[ref90] Yu-Cheng A. N. Shi-Fei T. Duan W. Qin Z. Hai-Long S. U. (2020). MiR-16-5p targets TXNIP to regulate LPS-induced oxidative stress and apoptosis in cardiomyocytes. Chin. J. Biochem. Mol. Biol. 36, 934–944. doi: 10.13865/j.cnki.cjbmb.2020.05.1452

[ref91] Zaghloul N. Kurepa D. Bader M. Y. Nagy N. Ahmed M. N. (2020). Prophylactic inhibition of NF-κB expression in microglia leads to attenuation of hypoxic ischemic injury of the immature brain. J. Neuroinflammation 17:365. doi: 10.1186/s12974-020-02031-9 33261624 PMC7709340

[ref92] Zarubin T. Han J. (2005). Activation and signaling of the p38 MAP kinase pathway. Cell Res. 15, 11–18. doi: 10.1038/sj.cr.7290257 15686620

[ref93] Zhang L. Fang Y. Zhao X. Zheng Y. Ma Y. Li S. (2021). miR-204 silencing reduces mitochondrial autophagy and ROS production in a murine AD model via the TRPML1-activated STAT3 pathway. Mol. Ther. 24, 822–831. doi: 10.1016/j.omtn.2021.02.010, PMID: 34026326 PMC8121631

[ref94] Zhang H. M. Kuang S. Xiong X. Gao T. Liu C. Guo A. Y. (2015). Transcription factor and microRNA co-regulatory loops: important regulatory motifs in biological processes and diseases. Brief. Bioinform. 16, 45–58. doi: 10.1093/bib/bbt085, PMID: 24307685

[ref95] Zhang X. Li R. Qin X. Wang L. Xiao J. Song Y. (2018). Sp1 plays an important role in vascular calcification both in vivo and in vitro. J. Am. Heart Assoc. 7:e007555. doi: 10.1161/JAHA.117.007555, PMID: 29572322 PMC5907546

[ref96] Zhang Z. B. Tan Y. X. Zhao Q. Xiong L. L. Liu J. Xu F. F.. (2019). miRNA-7a-2-3p inhibits neuronal apoptosis in oxygen-glucose deprivation (OGD) model. Front. Neurosci. 13:16. doi: 10.3389/fnins.2019.00016, PMID: 30728764 PMC6351497

[ref98] Zhao Y. Wang C. He W. Cai Z. (2022). Ameliorating Alzheimer's-like pathology by minocycline via inhibiting Cdk5/p25 signaling. Curr. Neuropharmacol. 20, 1783–1792. doi: 10.2174/1570159X19666211202124925, PMID: 34856907 PMC9881058

[ref99] Zhu K. Zhu X. Sun S. Yang W. Liu S. Tang Z. (2021). Inhibition of TLR4 prevents hippocampal hypoxic-ischemic injury by regulating ferroptosis in neonatal rats. Exp. Neurol. 345:113828. doi: 10.1016/j.expneurol.2021.113828, PMID: 34343528

[ref100] Zhuang X. Zhan B. Jia Y. Li C. Wu N. Zhao M.. (2022). IL-33 in the basolateral amygdala integrates neuroinflammation into anxiogenic circuits via modulating BDNF expression. Brain Behav. Immun. 102, 98–109. doi: 10.1016/j.bbi.2022.02.019, PMID: 35181439

